# The Photodynamic Bone Stabilization System in the Treatment of Humerus Fractures: A Prospective Clinical Trial

**DOI:** 10.7759/cureus.2809

**Published:** 2018-06-14

**Authors:** Paul Vegt, Jan Verbruggen, Thomas Gausepohl, Jos P Vroemen, Walter Schafer, Dietmar Pennig, Jeffrey M Muir

**Affiliations:** 1 Surgery, Albert Schweitzer Hospital, Dordrecht, NLD; 2 Surgery, University Hospital Maastricht, Maastricht, NLD; 3 Trauma Surgery, Lahn-Dill Kliniken, Klinikum Wetzlar, Marl, DEU; 4 Surgery, Amphia Hospital, Breda, NLD; 5 Clinic for Orthopedics, Kreiskrankenhaus Gummersbach, Gummersbach, DEU; 6 Surgery, St. Vinzenz Hospital Gmbh, Cologne, DEU; 7 Clinical Research, Telos Partners, Llc., Toronto, CAN

**Keywords:** long bone fracture, internal fixation, polymer, photodynamic bone stabilization system

## Abstract

Background

Fractures of the proximal humerus are among the most common long-bone fractures and present unique challenges to surgeons. Traditional internal fixation methods, such as intramedullary nails, are associated with drawbacks such as an inability to fully fill the trabecular space and a limited ability to utilize supplemental hardware in many cases. We evaluated the safety and effectiveness of a novel fracture fixation device that utilizes a light-cured monomer to stabilize the fracture in a cohort of patients suffering from humerus fractures.

Methods

We prospectively collected data from patients being treated surgically for humerus fractures. Fractures were treated using the photodynamic bone stabilization system (PBSS) consisting of a balloon and light-cured monomer. Patients were evaluated at 7-14, 30, 60, 90, 180, and 360 days post-procedure. Primary outcomes included normal and complete radiographic fracture healing. Secondary outcomes included pain (via visual analog scale), function (via the disability of the arm, shoulder, and hand (DASH) and constant shoulder scales), and the rate of complications.

Results

A total of 33 patients were included in the intent-to-treat analysis (mean age: 76.6 yrs). Of these patients, 88% demonstrated normal radiographic healing at their 90-, 180-, and 360-day visits. Complete radiographic healing was observed in 81%, 88%, and 96% of patients at 90, 180, and 360 days, respectively. Pain scores decreased significantly at day seven when compared with baseline (28.2+20.9 vs.53.6+32.2, p<0.001) and continued to decrease at the 90-day (24.7+15.5, p<0.001), 180-day (17.8+12.5, p<0.001) and 360-day (6.6+6.7, p<0.001) evaluations. DASH scores demonstrated statistically significant improvements over baseline (65.5+31.5) at 90 (37.0+14.9, p<0.001), 180 (30.6+15.7, p<0.001), and 360 days (23.9+15.0, p<0.001) post-procedure. The procedure-related event rate was 36.4%, with 5 (11.4%) device-related adverse events reported at the one-year follow-up.

Conclusions

Our study demonstrates the ability of a novel internal fixation device to safely and effectively treat fractures of the humerus in the elderly population.

## Introduction

Proximal humeral fractures are the second-most common fracture of the upper extremity and account for approximately 5% of all upper extremity fractures [1]. In patients aged 65 or older, humeral fractures rival only hip and wrist fractures in incidence and account for 10% of all fractures in this population [1-2]. Humeral shaft, distal humerus, and diaphyseal fractures are less common than proximal humerus fractures, although diaphyseal fractures constitute 20% of all humeral fractures [3]. While some proximal humerus fractures can be managed conservatively, many require surgical intervention for proper treatment [2].

Long bone fractures are traditionally treated surgically via either minimally invasive plate osteosynthesis (MIPO) or open reduction and internal fixation (ORIF), which utilizes intramedullary (IM) nails or plating to provide stability, although the use of IM nails has decreased somewhat in recent years [4]. While these methods provide sufficient fixation of the fracture, each is associated with significant drawbacks. Indeed, a recent systematic review and meta-analysis demonstrated that ORIF was associated with a higher rate of complication when compared with other methods, while MIPO was found to be associated with a lower rate of complications and better clinical outcomes [5]. In addition, the traditional treatment of long bone fractures–especially those involving internal fixation–are associated with the added drawback of being limited in their ability to completely fill the medullary space, thus threatening the overall stability of the construct. This lack of contact with the cortical wall can compromise the rotational stability of the bone and often necessitates the use of supplemental hardware, such as locking screws, to provide the required stability [6-8]. Drawbacks such as these contribute significantly to the challenges associated with traditional fixation methods for long bone fractures and are accentuated in patients with compromised bone density.

The recent development of an alternate fixation method–a photodynamic bone stabilization system (PBSS) that utilizes an intramedullary balloon and a light-cured monomer to provide fracture stabilization–provides the potential for improved fixation without the drawbacks of traditional methods. This device has been evaluated in clinical studies and has shown an excellent ability to stabilize long bone fractures [9-10]. The system provides an implant that is characterized by a malleable nature and a modulus of elasticity similar to that of bone, thus assisting with callus formation and promoting healing [11-12]. This system has been documented to successfully treat fractures of the tibia, fibular, and radius [9-10], with excellent clinical outcomes and rates of healing. The purpose of this study was to evaluate the safety and effectiveness of this system in the treatment of humeral fractures.

## Materials and methods

Study design

This study was a prospective, multicenter, open-label clinical study to evaluate the efficacy of the PBSS in patients with a fracture of the humerus. The primary objective of the study was to evaluate the safety and clinical performance of the device in the selected population. Ethics approval was received from all participating institutions prior to the commencement of the study. All patients provided informed consent for inclusion in the study.

Treatment intervention

The indications for use and the surgical procedure for the photodynamic bone stabilization system have been described elsewhere [9]. Briefly, the device consists of an intramedullary balloon and a fiber optic light pipe and its associated control technology. Following the reduction and stabilization of the fracture, an 8 mm percutaneous incision is used to insert the balloon into the intramedullary space. The balloon is positioned across the fracture, providing both longitudinal and rotational stability, due to its ability to fully contact the cortical wall. Once positioned appropriately, the balloon is infused with a biocompatible photodynamic liquid monomer and, after proper alignment is ensured, the fiber optic light pipe is inserted through the inner lumen of the balloon. The light pipe is controlled by the surgeon using an external console and timer key that quickly polymerizes the liquid, forming a strong, hardened bone-stabilizing rod. The monomer requires between 200 and 800 seconds for hardening, depending on volume. Once polymerized, the construct provides structural stability but also allows for the use of osteosynthesis hardware as needed, as the hardened monomer provides an excellent substrate into which screws can be inserted.

Patient eligibility

This study included a population of skeletally mature adult patients with a single, acute, isolated humerus fracture. Specific inclusion and exclusion criteria included are as follows:

Inclusion criteria: Patients were eligible for inclusion in the study if they fulfilled all of the following inclusion criteria:

- Humerus fracture, AO classification type: 11A (1, 2, or 3), 11B (1 or 2), 11B2, 12A (1, 2, or 3), 12B (1 or 2), 13A (1 or 2) or 13B (1 or 2) (Germany only), 11A (2 or 3), 11B (1 or 2), 12A (1, 2, or 3), 12B (1 or 2) (The Netherlands only), or closed fracture, Gustilo Type I or II,

- Skeletally mature adult, 50 years of age or older (The Netherlands only) or 61 years of age or older (Germany only) at the time of index injury, and

- Ability and willingness to understand and sign the informed consent form.

For female patients who were potentially eligible for inclusion, additional criteria included:

- For patients of child-bearing age, agreement to using the double barrier method of contraception (The Netherlands only), or

- For patients of non-child-bearing age, the potential for one of the following (The Netherlands only):

o Post-menopausal for at least one year,

o Documented oophorectomy or hysterectomy, or

o Surgical sterility.

Exclusion criteria: Patients were not eligible for inclusion if they fulfilled any of the following:

- Index treatment occurred for greater than 28 days (The Netherlands only) or 14 days (Germany only) post-fracture,

- Open fracture with severe contamination,

- Extremely comminuted fracture where an insufficient holding power of the balloon on the medullary canal was probable,

- Marked bone loss, bone resorption, prior delayed, non-union of bone or other illnesses that would prevent adequate reduction of the fracture prior to the placement of the PBSS (i.e., cases where the potential for bone void space(s) that would preclude the device from maintaining alignment of the fractured bone existed), or

- Previous fracture of the affected limb.

Additional general exclusion criteria are summarized in Table 1.

**Table 1:** General exclusion criteria

General exclusion criteria
Pregnant or lactating female patients (The Netherlands only)
Patients with a contralateral fracture of the forearm or humerus (Germany only)
Patients with active or incompletely treated infections that could have involved the site where the device was to be implanted
Patients with distant foci of infection that could spread to the implant site
Uncooperative patients or patients who were incapable of following directions (e.g. as a consequence of a neurological or psychiatric disorder)
Patients with concomitant metabolic disorders that could have impaired bone formation
Patients with osteomalacia
Patients who were allergic to implant materials or dental glue
Patients with vascular insufficiency, muscular atrophy or neuromuscular disease
Polytrauma patients (multiple injuries resulting from a high impact event, e.g. a motor vehicle accident)
Patients with a life expectancy less than one (1) year due to concurrent illness

Patient allocation and schedule

As this study prospectively enrolled patients based on eligibility status, there was no blinding of patients or surgeons. Patients who fulfilled all of the inclusion criteria, none of the exclusion criteria, provided informed consent, and agreed to participate were enrolled in the study consecutively. 

The study included eight follow-up appointments, including screening/baseline visit, surgical visit, post-index procedures, discharge, and follow-up appointments, which occurred at 7-14, 30, 60, 90, 180, and 360 days post-procedure (Table 2). Patients were deemed to have fully completed the study following their one-year (360-day) follow-up.

**Table 2:** Summary of outcome data collected at follow-up appointments

	Visit 1	Visit 2	Visit 3^1^	Visit 4	Visit 5	Visit 6	Visit 7	Visit 8
	Screening & Baseline	Surgery & Discharge	7-14 Day F/U (+ 3 Days)	30 Day F/U (± 14 Days)	60 Day F/U (± 14 Days)	90 Day F/U (± 14 Days)	180 Day F/U (± 30 Days)	360 Day F/U (± 60 Days)
Informed Consent	X							
Medical History	X							
Physical Exam	X							
Clinical Assessments^2^			X	X	X	X	X	X
Disability of the Arm, Shoulder, and Hand (DASH) Score^3^	X		X	X	X	X	X	X
Pain Visual Analog Scale (VAS)	X		X	X	X	X	X	X
Constant Shoulder Score	X		X	X	X	X	X	X
Total Active & Passive Range of Motion			X^4^	X^4^	X^4^	X^4^	X^4^	X^4^
Radiograph of Fracture	X		X			X	X	X
Adverse Events		X	X	X	X	X	X	X

Study outcomes

The primary outcome of this study was fracture healing at the injury site at six-months post-procedure, assessed as both normal healing and complete healing via radiographs. Normal radiographic healing was defined by two of four cortices or two of four views demonstrating bridging on standard radiographs, while complete radiographic healing was defined as three or four cortices or three of four views demonstrating bridging with the dissolution of the majority (≥75% on orthogonal views) of fracture lines. The primary endpoint was considered to have been met if a minimum of 90% of patients were deemed to exhibit normal fracture healing at the day-180 evaluation.

Secondary outcomes evaluated at each follow-up visit included: pain (via visual analog scale, VAS); upper limb functional abilities, via the disability of the arm, shoulder, and hand (DASH) survey; constant shoulder score; disability status, as determined by Investigator assessment; and return to work status (where applicable). An assessment of the incidence of adverse events and an assessment of the procedure- and device-related complication rate was made at the six-month and one-year follow-up visits. 

Statistical analysis

Statistical analyses were completed per a statistical analysis plan, with alpha set a priori at 0.05 for statistical significance. Results are presented for the intent-to-treat (ITT) population. Means are presented as mean (standard deviation) for continuous variables and as percentages for categorical variables. Values were compared using the dependent and independent sample t-test, analysis of variance (ANOVA), Fisher’s exact test, and chi-squared test, where appropriate.

A minimum clinically important difference (MCID) is the change in a scaled value that is considered to be clinically relevant. For the VAS, a change of between 1.37 on a 10-point scale [13] to 30 points on a 0-100 scale [14] have been suggested as clinically relevant. For the purposes of this study, we determined that a change of 20 points on a 100-point scale would constitute the minimum clinically important difference. Statistical analysis was completed using SAS for Windows, Version 9.2 (Cary, NC, USA).

## Results

Study population and patient demographics

A total of 44 patients signed the informed consent form and were enrolled in the study. Of these, 33 fulfilled all of the inclusion criteria and constituted the intent-to-treat (ITT) population. Thirty-two patients completed the day 180 visit for the primary endpoint; full one-year follow-up data is available for 37 patients. Demographic statistics describing the ITT population are summarized in Table 3. The ITT population had a mean age of 76.6 years (range: 69-98) and was 75.8% female (25/33). Of these, 93.9% (31/33) of the patients were not working at the time of their surgery. Seven patients (21.2%) had a confirmed radiographic diagnosis of osteoporosis at the time of their inclusion in the study.

**Table 3:** Demographic statistics for the intent-to-treat (ITT) population

Characteristic	Result (n=33)
Age, years: mean (SD, range)	76.6 (10.2, 69-98)
Gender, female: n (%)	25 (75.8)
Work status: n (%)	
Not working at time of fracture	31 (93.9)
Full time	1 (3.0)
Part time	1 (3.0)
Restricted duty	0 (0)

The left humerus was the location of the fracture in 23 patients (69.7%), while the vast majority of patients (32/33, 97%) suffered fractures in the proximal humerus. Fracture characteristics are further summarized in Table 4. Twelve fractures (36.4%) were traumatic in nature, with 20 (60.6%) considered non-traumatic but associated with a low-energy fall. One fracture was non-traumatic and not associated with a fall. Mean procedural time was 1.5 hrs (SD: 0.52, range: 0.78-2.75) while the mean length of stay (LOS) was 10.3 days (SD: 7.1, range: 1-25).

**Table 4:** Fracture characteristics

Characteristic	Result (n=33)
Location of target fracture, n (%)	
Left humerus	23 (69.7)
Right humerus	10 (30.3)
Location within humerus, n (%)	
Proximal	32 (97.0)
Diaphyseal	1 (3.0)
Distal	0 (0)
AO classification	
02-A3	0 (0)
11-A1	1 (3.0)
11-A2	8 (24.2)
11-A3	2 (6.1)
11-B1	9 (27.3)
11-B2	13 (39.4)
11-C2	0 (0)
Gustilo Grading of Soft Tissue, n (%)	
Closed	26 (78.8)
Type I	7 (21.2)
Type II	0 (0)
Type III A	0 (0)
Type III B	0 (0)
Type III C	0 (0)
Neer's Fracture Grading, n (%)	
N/A	4 (12.1)
2-part	11 (33.3)
3-part	14 (42.4)
4-part	4 (12.1)
Type of injury, n (%)	
Traumatic	12 (36.4)
Non-traumatic, low energy fall	20 (60.6)
Non traumatic	1 (3.0)
Unknown	0 (0)
If traumatic, specify	
Motor vehicle accident	0 (0)
Sports injury	2 (6.1)
Work injury	0 (0)
High energy fall	0 (0)
Other	10 (30.3)

Fracture healing

In the ITT population, the primary effectiveness outcome was realized in 88% (27/28) of patients with a valid assessment, with this group demonstrating normal radiographic healing of the index fracture at each of their 90-, 180-, and 360-day appointments (95% CIs: (0.761, 1.000), (0.887, 1.000), and (0.895, 1.000), respectively). Complete radiographic fracture healing was observed in 81% (21/26, 95% CI: (0.656, 1.000)) of patients with a valid assessment at 90 days, 88% (23/26, 95% CI: (0.761, 1.000)) of patients at 180 days and 96% (27/28, 95% CI: (0.895, 1.000)) at one year.

For patients who met the primary effectiveness endpoint, a subgroup analysis was performed, comparing patients treated with PBSS alone or PBSS plus additional hardware (plates, screws). Twenty-three (69.7%) patients were treated with PBSS alone or with the addition of one or more screws. Sixteen of 23 patients with PBSS alone or with screws had a valid assessment, and of those, 100% demonstrated normal radiographic healing at their 180-day visit versus 90% (9/10) of patients whose treatment was augmented with plates (p=0.95). Complete radiographic healing was observed in 100% of patients with screws and in 70% (7/10) of patients with PBSS plus plating (p=0.95).

Pain scores

**Figure 1:**
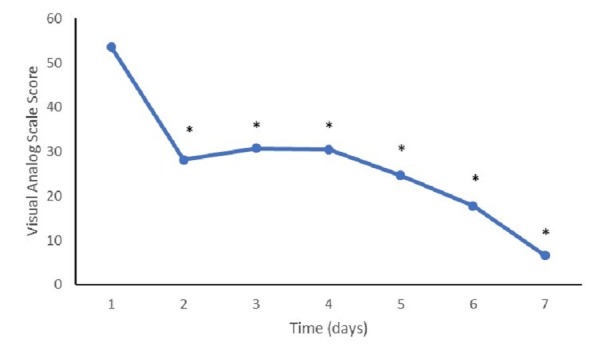
Visual analog scale at follow-up Mean visual analog scale scores at study follow-up appointments. (*) represents a value significantly different from the baseline (p<0.05).

The mean pain (VAS) score at baseline was 53.6 (SD: 32.2), which improved significantly within the first seven days post-surgery, an improvement that was maintained through one-year follow-up (Figure 1). The mean VAS score at the seven-day visit was 28.2 (SD: 20.9), a 47% improvement over baseline scores (p<0.001). Mean VAS scores remained significantly decreased compared to the baseline at 90 days (24.7, SD: 15.5, p<0.001), 180 days (17.8, SD: 12.5, p<0.001) and one year (6.6, SD: 6.7, p<0.001).

A minimum clinically important difference (MCID) versus the baseline in VAS scores was noted in 71% (12/17) of the patients at both the 90- and 180-day follow-up visits. At one year, 82% (14/17) of patients realized an MCID in VAS scores versus the baseline.

Functional abilities

**Figure 2:**
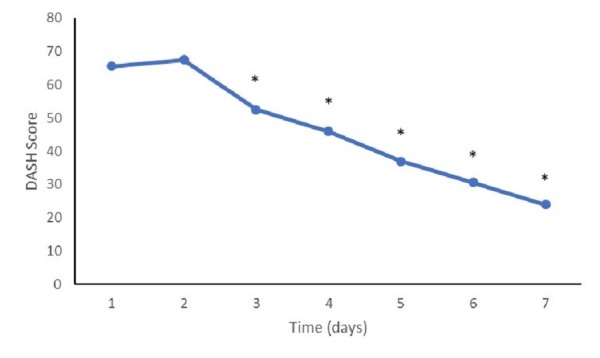
DASH scores at follow-up Mean disability of the arm, shoulder, and hand (DASH) survey scores at follow-up appointments. (*) represents a value significantly different from the baseline (p<0.05).

**Figure 3:**
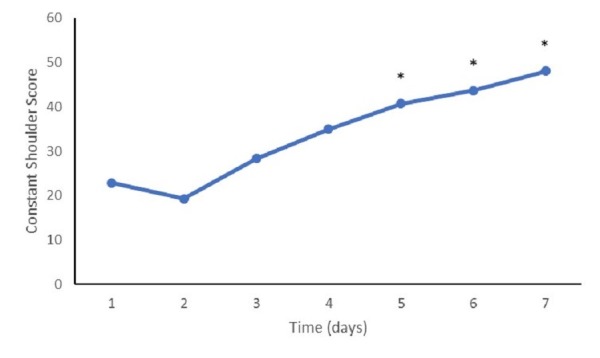
Constant shoulder scores at follow-up Mean constant shoulder survey scores at follow-up appointments. (*) represents a value significantly different from the baseline (p<0.05).

The mean baseline DASH score was 65.5 (SD: 31.5). The mean DASH scores at 90 days (37.0, SD: 14.9, p<0.001), 180 days (30.6, SD: 15.7, p<0.001), and one year (23.9, SD: 15.0, p<0.001) evaluations had all improved significantly when compared with the baseline scores (Figure 2). Constant shoulder scores also demonstrated similar improvement from baseline scores (22.9, SD: 25.4), with statistically significant improvements noted at the 90-day (40.3, SD: 14.1, p=0.001), 180-day (43.7, SD: 16.5, p=0.0002), and one-year (48.1, SD: 14.9, p<0.001) follow-up visits (Figure 3).

Complications

The procedural complication rate was 36.4%. The most commonly reported complications were wound secretion and pain, both of which occurred in four (9%) patients. In the 44 patients initially enrolled in the study, five (11.4%) device-related adverse events (DRAEs) were reported. Device-related events included device breakage (one), device dislocation (one), delayed fracture union (one), and dislocation of device screw (two). These events were deemed “possibly related” to the device by the investigator. No DRAEs were deemed to be “definitely related” to the device and no DRAEs were considered severe.

## Discussion

Proximal humerus fractures are among the most common upper extremity fractures and are generally treated surgically using traditional methods such as open reduction and internal fixation (ORIF). While associated with good results, the use of internal fixation, such as intramedullary (IM) nails, is associated with drawbacks such as higher complication rates. A novel fixation system, the photodynamic bone stabilization system, has recently been developed and provides excellent stability without the drawbacks associated with traditional internal fixation. We evaluated the safety and effectiveness of this novel system in a population of patients suffering from humerus fractures and observed excellent healing at mid- and long-term follow-ups and significant decreases in pain. This system may offer a viable alternative for surgeons seeking to stabilize upper extremity fractures.

In our study, we observed excellent rates of fracture healing at 90-days, 180-days, and one-year post-surgery. Several studies investigating the surgical treatment of proximal humerus fractures have observed similar results [15-25], but there are several differences with our study. Primarily, our study population is older than in the majority of other studies. As a result, the likelihood of comorbidities and the additional challenges associated with fracture healing in an older population are present. Nevertheless, we observed fracture healing rates of 96%, a finding that was consistent through all follow-up visits, up to one-year post-procedure. In previous studies, the PBSS system has been associated with similar rates of healing in osteoporotic fractures, with progressive or complete radiographic healing noted in 96% of patients at one year [10] and equally high rates of healing in a population of elderly patients suffering from fragility fractures [9]. The clinical results from this and other studies involving this system have also highlighted the ability of the system to fully fill the intramedullary space [9], thus providing superior stability when contrasted with other internal fixation devices, such as IM nails, which do not fill the cavity completely. This increased stability provides an ideal construct for bones of compromised structural integrity, as is the case in an elderly and potentially osteopenic population such as that treated in this study.

The increasing incidence of low bone density among the aging population has resulted in an increased need for and a reliance on supplemental hardware when surgically treating long bone fractures [26]. The use of traditional methods of fixation such as IM nails limits this ability to use supplemental hardware. In addition to the aforementioned drawback of not completely filling the intramedullary space, the limitations associated with supplemental hardware add to the challenges faced by surgeons when treating long bone fragility fractures in an elderly population. The ability of the PBSS device to provide a substrate for the insertion of additional screws and/or plates positions it as a potentially valuable treatment option for this population. Indeed, 30% of our population had their implant augmented by plates and screws. At 180 days, 100% of these patients demonstrated normal and complete radiographic healing, indicating no difference between the rates of radiographic healing with those patients who did not require supplementary plating (p=0.95 for both normal and complete radiographic healing). The flexibility in selecting treatment options that these findings provide to surgeons treating similar fractures in osteopenic and/or osteoporotic populations is an important benefit of the PBSS device and one that is not available with traditional fixation methods.

Our study reported a procedural complication rate of 36.4%, with an 11.4% rate of device-related adverse events. These results compare favorably with those of other authors. In a recent retrospective study, Repetto et al. [27] reported a 31.5% complication rate and a rate of revision surgery of 14.1%. Similarly, Lange et al. [28] prospectively compared surgical treatment with conservative care and observed a complication rate of 37% in the surgical group but also observed a high rate of reoperation (32%). Finally, during a systematic review of IM nailing for proximal humerus fractures, Wong et al. [29] analyzed the results of 14 clinical trials and found an overall complication rate of 41.5%. That our study reported a rate of complications on par with these findings is an important observation. The most common complications reported in our cohort were wound secretion and pain, both of which occurred in 9% of the population. Device-related complications also occurred at a rate comparable to traditional internal fixation methods [29]. This safety profile has been observed previously with the PBSS device, with comparable rates of complications observed retrospectively [9-10]. The observation of a similar rate of complications in prospectively collected data is an important observation and one that indicates the overall safety of the device when compared with the standard of care for long bone fracture fixation.

Our study is not without limitations. Primarily, the non-randomized nature of patient enrollment could limit the veracity with which the conclusions can be applied to other populations. However, the prospective nature of the study, in addition to the intent-to-treat analysis, represent valid and important methodological factors for this study. We have previously demonstrated the efficacy of this system in retrospective, prospective, and registry studies [9-10]. The addition of this study, with data collected prospectively, improves the overall quality of the evidence in the literature. A second limitation of this study could be the relatively small sample size; however, the results of this study demonstrate consistency with previous studies of this device [9-10], all of which also involved relatively small sample sizes. Also, the recent publication of a summary of the European Registry, a cohort of 132 patients and 149 treated fractures [10], provides evidence from a larger patient cohort, with results similar to those observed in this study. While prospectively collected data in a randomized study is needed, the data in this study provides important evidence in support of PBSS.

## Conclusions

Our study demonstrated the ability of a novel internal fixation device to promote the healing of humerus fractures in an older patient population. The ability of the device to fully fill the intramedullary space and provide a solid construct that provides both rotational and longitudinal stability while allowing for the use of supplementary hardware is an important observation. While further research is required to further evaluate this novel stabilization system, these results are promising and indicate a potentially major role for this technology moving forward.
